# Positioning Performance of Power and Manual Drivers in Posterior Spinal Fusion Procedures

**DOI:** 10.1155/2017/7262841

**Published:** 2017-07-27

**Authors:** J. Micah Prendergast, Alexander C. Perry, Vikas V. Patel, Emily M. Lindley, Mark E. Rentschler

**Affiliations:** ^1^Department of Mechanical Engineering, University of Colorado at Boulder, 427 UCB, Boulder, CO 80309, USA; ^2^University of Colorado Anschutz Medical Campus, Aurora, CO, USA

## Abstract

This work presents an analysis and comparison of the efficacy of two methods for pedicle screw placement during posterior spinal fusion surgery. A total of 100 screws (64 manual and 36 power driven), all placed utilizing a surgical navigation system, were analyzed and compared. Final screw placement was compared to initial surgical plans using the navigation system, and the final screw locations were analyzed on the basis of angular deviation from these planned trajectories as well as screw translation within a critical reference plane. The power driver was found to insignificantly decrease the resulting angular deviation of these pedicle screws with a mean deviation of 3.35 degrees compared to 3.44 degrees with the manual driver (*p* = 0.853). Conversely, the power driver was found to increase the translational distance in the critical region, with mean deviations of 2.45 mm for the power driver compared to 1.54 mm with the manual driver. The increase in translational deviation was significant (*p* = 0.002) indicating that there may be some loss in performance from the adoption of the power driver.

## 1. Introduction

Spinal fusion procedures are used to treat a variety of spinal conditions, including injuries to the vertebrae, spinal instability due to infections or tumors, and abnormal spinal curvature. Spinal fusions with instrumentation are typically able to relieve pain in the lower back and lower extremities and correct abnormal spinal geometry [1, 2]. The presence of nervous tissue in and around the spinal vertebrae, however, makes this procedure more prone to complications when compared to other orthopedic surgeries [3]. Given this concern, the accurate placement of pedicle screws is essential. A variety of surgical tools have been designed to aid surgeons in these procedures and to improve insertion accuracy to avoid serious complications. These tools include navigation systems that allow for virtual visualization of the spine geometry and screw trajectories and power-driven screwdrivers to decrease procedure time and lessen surgeon fatigue during procedures. This work presents a detailed analysis of the results of using one such power screwdriver (POWEREASE™, Medtronic Inc., Louisville, CO) in tandem with a virtual navigation system (StealthStation S7, Medtronic Inc., Louisville, CO).

## 2. Background and Motivation

### 2.1. Posterior Spinal Fusion

Posterior spinal fusion (PSF) is a procedure in which bone growth is promoted between two or more vertebrae, eventually joining them into a single bone. This procedure may be performed for a variety of reasons, including damage to the spine such as degenerative changes, fracture or damage to an intervertebral disc, spinal deformations such as scoliosis or kyphosis, spinal instability due to infection or tumor removal, or relief of pain due to abnormal bone growth as in spinal stenosis [1, 2].

**Figure 1:**
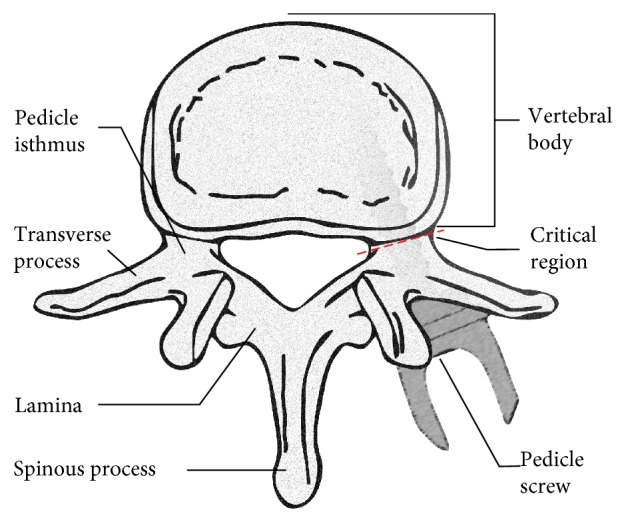
Transverse cross section of vertebra. In spinal fusion procedures, pedicle screws are inserted through the pedicle and into the vertebral body.

**Figure 2:**
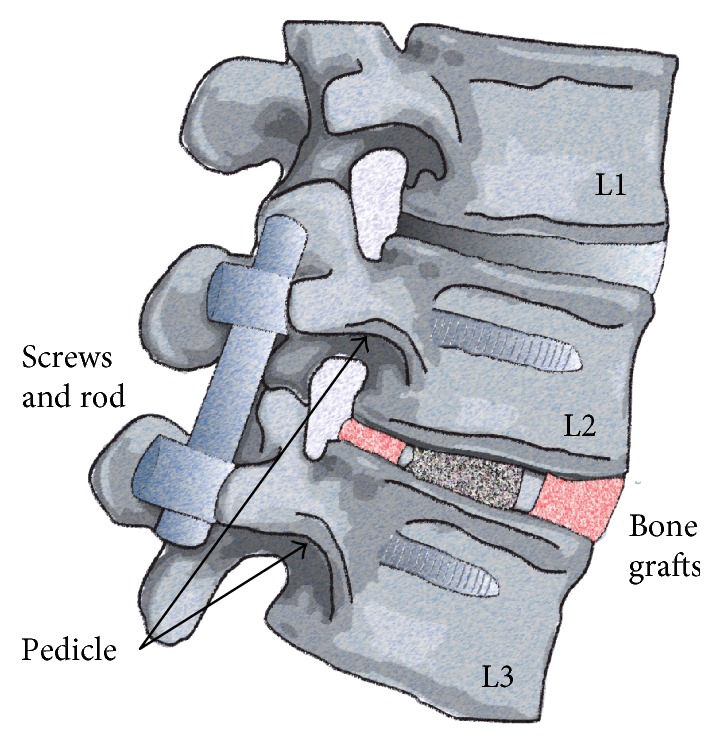
Pedicle screws and support rods are shown in this image. Also note the location of the bone graft between vertebrae.

During a PSF procedure, pedicle screws are inserted into the vertebra pedicle (Figure 1) to allow for the attachment of connecting rods. These rods serve to stabilize or fix the spine to facilitate bone growth (Figure 2).

Due in part to the critical nature of the spine and the nervous tissue protected by the spinal column, PSF procedures involve a range of risks, including the potential for nervous tissue damage. In particular, complications may occur due to inaccurate pedicle screw placement that results in a medial breach or perforation of the pedicle. In addition, poor screw placement may allow for premature loosening of screws, potentially resulting in a failure to fuse. For these reasons, significant emphasis is given to the careful training of surgeons in the correct placement of pedicle screws. Expert knowledge of the spinal anatomy and relevant anatomical landmarks in tandem with guided training and experience is paramount for surgeons to accurately place pedicle screws.

### 2.2. Spinal Navigation

While accurate pedicle screw placement can be achieved by skilled surgeons without the use of imaging or navigation tools [4, 5], a variety of navigation aids have been designed to assist surgeons and to increase the overall accuracy and success of spinal fusion procedures. Traditional fluoroscopy has been used for years to visualize the spine, both to locate and to account for abnormalities, and to assess final screw placement [6, 7]. More advanced techniques such as computed tomography (CT) navigation and fluoroscopic-based navigation allow for real-time visualization of the spine with respect to the surgeon's tools [8–10]. 3D navigation in particular has been shown to significantly improve accuracy in pedicle screw placement and to minimize medial breaching of the pedicle isthmus which most endangers nervous tissue [8, 9].

### 2.3. Power Driver

Even with the use of navigation aids for pedicle screw placement, spinal fusion procedures still pose significant technical challenges to surgeons and require intense physical effort from the surgeon throughout the procedure. In addition, as prolonged operative time has been shown to increase the risk of infection and other complications for many minimally invasive procedures, a reduction of operative time stands to significantly benefit patients [11–14].

Given the importance of minimizing operative time as well as overall surgeon effort during spinal fusion procedures, the use of power-assisted drivers is being adopted. The power driver used in this study has been designed to decrease surgeon fatigue during screw insertion. In addition, according to the manufacturer, this driver may offer significantly reduced procedure times, demonstrating a 51% reduction in the time required for the tapping of the pedicle and a 55% reduction in the time required for inserting pedicle screws as described in a study conducted by the manufacturer [15].

While the development of the power driver may aid in the advancement of PSF, to the authors' knowledge, no comparative accuracy study between a power and manual driver has been conducted. This work aims to provide quantitative data comparing the two methods of pedicle screw insertion.

## 3. Methodology

The study methodology is divided into five sections. The first section briefly describes the study design. The second section describes the collection of data using the StealthStation and O-arm imaging system (O-arm Surgical Imaging, Medtronic Inc., Dublin, IE) for both manual and power procedures. These systems are used for all patients throughout this study and use intraoperative 3D scans and optical tracking to provide pseudo-real-time feedback to the surgeon with regard to tool position relative to patient anatomy. The third section describes the postprocessing steps that were taken after the procedure to determine the final screw placement. Finally, the last two sections detail the two metrics used to assess procedure performance: angular deviation and reference point deviation. All research was conducted in accordance with a test protocol approved by the University of Colorado Anschutz Medical Campus's Institutional Review Board (COMIRB Protocol 11-0990).

### 3.1. Study Design

After local IRB approval, a consecutive series of patients undergoing posterior spinal fusion were prospectively recruited, and those agreeing to participate were consented for inclusion in the study. Patients meeting the following inclusion criteria were eligible for enrollment: (1) aged 18–75, (2) scheduled to undergo thoracic and/or lumbar posterior spinal fusion procedures with pedicle screw placement using O-arm and StealthStation at the University of Colorado Hospital, and (3) planned fusion involved at least 2 motion segments. Exclusion criteria were (1) pregnancy and (2) revision surgeries with previous pedicle screw instrumentation. All procedures were performed by two surgeons with more than 10 years of clinical practice, including regular use of navigation during pedicle screw placement. The power driver was available to the surgeons for a limited time during the study, so all subjects enrolled during that time period had screws placed with the power driver. Patients enrolled outside of that time frame had pedicle screws placed with a manual driver.

### 3.2. Surgical Procedure

**Figure 3:**
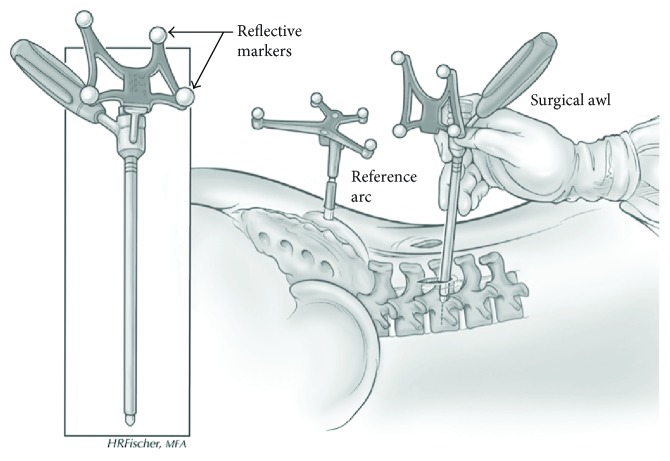
Schematic of reference arc attached to patient. Reflective optical markers are appended to each tool to facilitate localization by the navigation system. *Image is reproduced from an open access journal* © Park et al. [16].

Once general anesthesia was administered to the patient, access to surgical space was achieved via open incision. A reference arc is rigidly attached to one of the patient's vertebra (typically on the spinous process of one of the vertebrae to be included in the fusion) and the imaging system is moved into position to take the first 3D scan with the 3D camera system in place. This scan captures the spinal region to be fused and locates the reference arc with respect to the patient's spine (Figure 3).

Following the reference arc attachment and initial 3D scan, tool positions and orientations are tracked via the surgical navigation system and overlaid onto the initial 3D scan. This visualization allows for pseudo-real-time navigation of the tools with respect to the patient's anatomy with an accuracy of approximately 1 to 1.4 mm [17–19]. It should be noted that for all patients, screws were placed first after exposure while the spine was still stable to minimize error from the motion of the spinal segments that are further from the reference frame.

**Figure 4:**
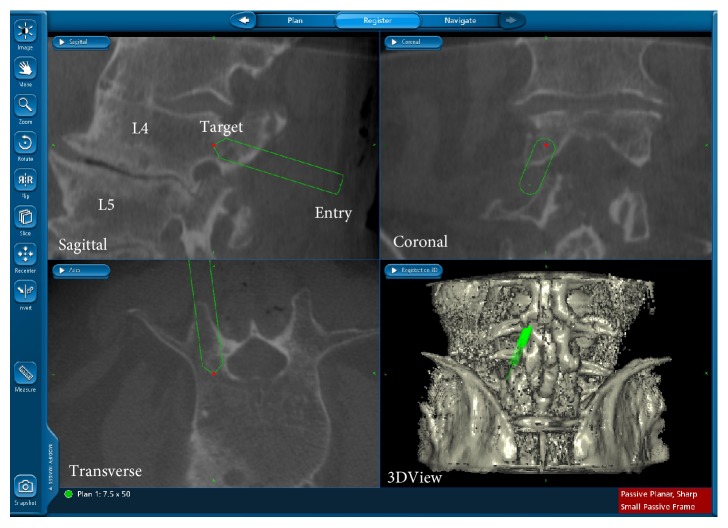
Screenshot taken from the StealthStation navigation system. The display that a surgeon has access to during a procedure is shown. The location of each vertebra, the screw trajectory coordinates, and the anatomical planes are marked in this figure, but are not during the procedure. This image was obtained from the workstation's spinal software.

Before each screw is inserted, several initial steps are taken by the surgeon. First, a surgical awl is inserted into the pedicle. The awl, which is tracked via the navigation system, is positioned with its tip at a depth within the pedicle isthmus which the surgeon believes most susceptible to a medial breach. This initial “Entry” trajectory depth position is saved by the technician operating the navigation system (Figure 4). Once the “Entry” trajectory depth has been saved, the surgeon continues to insert the awl deeper, through the pedicle, into the vertebral body until the surgeon believes the awl is at a depth that will allow the screw to securely anchor upon insertion. A new trajectory is saved by the technician at this point as the “Plan” trajectory.

With these initial steps complete, and the Entry and Plan trajectories saved, the surgical awl is removed and screw insertion can begin (in rare instances of hard bone, nonnavigated tapping, up to a max depth of 20 mm was also performed). For each patient, all screws are inserted with either a manual- or power-assisted driver, with the surgeon using the same type of driver for all screws in a given patient. Both tools include passive reflective markers so they can be optically tracked by the navigation system throughout the insertion. When the screw is tightened into its final position, the trajectory of the driver is saved in the navigation system as the “Screw” trajectory. These steps, including the insertion of the awl and screw insertion, are then repeated for all subsequent screws.

After all screws have been inserted, a second 3D scan is taken which serves to capture the final locations of all screws relative to the reference arc. Once this scan has been completed and saved, the reference arc is removed. In total, 64 manually driven and 36 power-driven screws were inserted in 11 patients throughout this study.

### 3.3. Postprocessing and Determination of Actual Trajectory

Following each spinal fusion procedure, O-arm scans were examined to assess final screw placement. To do this, the two 3D scans (the first of the Plan and Screw trajectories and the second which shows the final screw positions) must be carefully aligned and merged using the second scan as the reference image. An initial merge of the scans is done automatically via StealthStation's Cranial Suite; however, this merge is not accurate enough to allow for good analysis, and it is not possible to export these images to 3rd party software for custom merging. Thus, a final, manual merge is completed by a single user trained by Medtronic in the correct operation of the StealthStation. This user must visually align the two scans until no discernable difference exists in any of the three imaging planes (coronal, sagittal, and transverse). Several tools, including zoom, color, and transparency adjustment, are built into the Cranial Suite to allow for the effective and accurate alignment and merging of the two scans. While all postprocessing was performed by a single user, no statistically significant difference was seen when comparing this primary user to a trained, secondary user.

**Figure 5:**
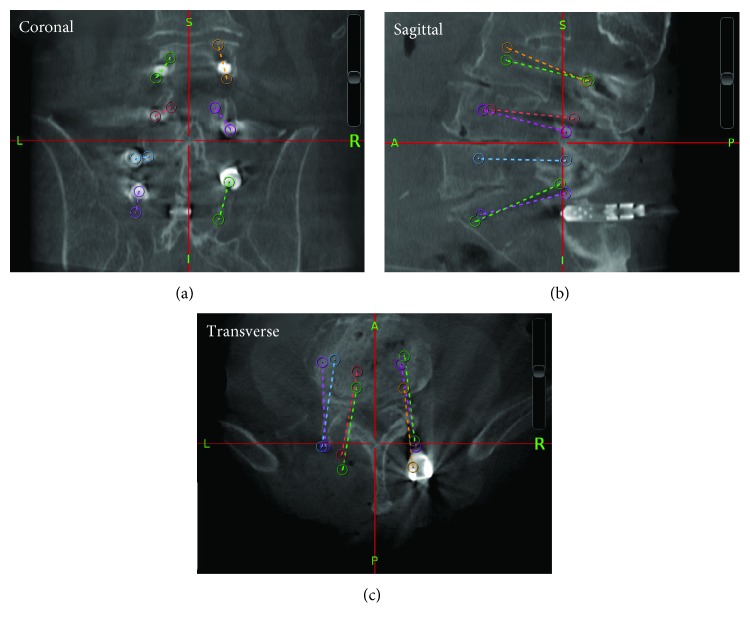
Actual trajectories obtained from the StealthStation (represented by the colored, dashed lines), which were drawn in the software by the workstation user. (a) shows the coronal frame, (b) shows the sagittal frame, and (c) shows the transverse frame. This image was obtained from the workstation's Cranial Suite software.

Once the scans have been merged, the final position of each screw must be drawn (Figure 5). This is done again utilizing the three image planes of the merged scan. Entry points are first drawn at the transition point between the head and the body of the screw as close to the center of the screw as can be visibly inspected. This position is important as the head of spinal screws is not fixed, and thus, attempting to define the measurements origin on the very end of the screw's head would likely lead to an inaccurate trajectory. Once the Entry point has been marked, the terminal point is marked at the screw's tip to create the final or “Actual” screw trajectory. The Cranial Suite, again, provides several useful tools for the drawing of these trajectories and individual points are visualized in all three planes at once as well as in a 3D view, so as to provide accurate positioning of each trajectory. Once all trajectories for a single merged scan have been drawn, the data representing these trajectories as well as the transformation matrix describing the rotational and translational shift required to merge the scans are exported to text files. Again, no statistically significant difference was found when comparing the trajectories drawn by the primary user to those drawn by a second trained user or when examining the primary user's analysis of the same data over multiple months.

When all data have been exported from the navigation system, they are imported into MATLAB (MathWorks, Natick, MA) for analysis. The Actual trajectory's entry and terminal points are multiplied by the transformation matrix corresponding to the merge, and all trajectories are then drawn and visualized in MATLAB to correlate imported data to StealthStation data and as a visual verification to ensure no unexpected errors occurred while drawing trajectories or exporting the data. At this point, all data are ready for final comparison and analysis.

### 3.4. Angular Deviation

A common metric used to assess the success of pedicle screw placement is the angular deviation between the final screw location (Actual trajectory) and the intended screw location (Plan trajectory). Given the assumption that the Plan trajectory and the Actual trajectory should have nearly identical entry locations, it is expected that translational deviations between trajectories will be very small. Thus, small angular deviations between these trajectories should be indicative of precise screw placement. As the initial hole created by the awl serves as the entry point for the screw, this assumption should be valid in most cases. It should also be noted that while the above does make the assumption that the vectors share a common origin, this does not affect the calculated angular deviation.

Once the Actual trajectory has been transformed into the Plan/Screw trajectory frame, the angular deviation between any two of the three trajectories can be calculated using (1), where *T*_*α*_ and *T*_*p*_ are vectors representing the two trajectories being compared.

(1) $$\begin{array}{c} \theta =\cos^{-1}\frac{T_{\alpha }\cdot T_{p}}{\left|T_{\alpha }\right|\left|T_{p}\right|}. \end{array}$$

In addition to calculating the angular deviation between the Actual and Plan trajectories, angular deviations were also calculated between the Actual and Screw trajectories as well as the Screw and Plan trajectories.

### 3.5. Reference Point Deviation

While the angular deviation calculation is a commonly used calculation for determining accurate screw placement, this calculation does not assess any translational differences that may occur between trajectories. More importantly, while angular deviation provides a useful metric for determining overall alignment of pedicle screws, it does not necessarily indicate how close these screws came to breaching the pedicle isthmus. Because a pedicle breach (especially a medial pedicle breach) represents a key failure criterion when assessing pedicle screw placement and because any movement of the patient or reference arc may result in unintended translational errors, an additional metric was employed to compare screw placement within a critical region of the pedicle isthmus (Figure 1).

The critical region is determined via the Entry trajectory that is recorded initially as the awl is pressed into the pedicle isthmus. This trajectory ends with the tip of the awl at a point which we will refer to as the critical depth (CD) within the isthmus. This point does not necessarily represent the center of the isthmus, but is estimated to be close to the depth within the isthmus where the risk of perforation is the highest.

**Figure 6:**
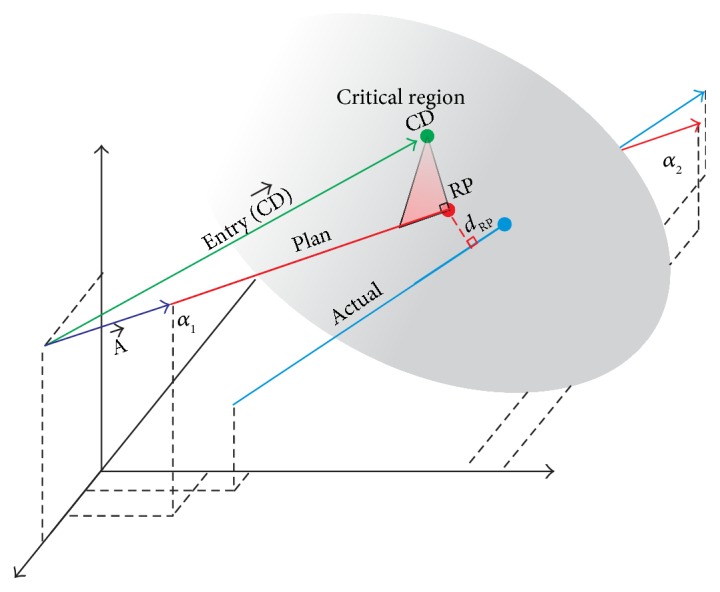
Visualization for determining the reference point deviation (*d*_RP_) of the actual trajectory from RP. Please note that both angular and reference point deviations are significantly exaggerated here for visualization purposes.

Given point CD, the closest point along the line defined by the Plan trajectory vector to CD can be obtained. This new point is used as the reference point (RP), and points RP and CD can then be used to define a plane within the pedicle which we will refer to as the critical region (Figure 6).

As shown in (2), vector algebra can be used to find RP. In this equation, $\vec{N}$ represents a unit vector in the direction of the Plan trajectory and $\vec{A}$ represents a vector pointing from the start of the Entry vector to the start of the Plan vector. Finally, vector $\vec{CD}$ represents a vector with direction and magnitude identical to the Entry trajectory, pointing at point CD.

(2) $$\begin{array}{c} RP=\left(\vec{A}-\vec{CD}\right)-\left[\left(\vec{A}-\vec{CD}\right)\cdot {\vec{N}}^{T}\right]\vec{N}+\vec{CD}. \end{array}$$

**Figure 7:**
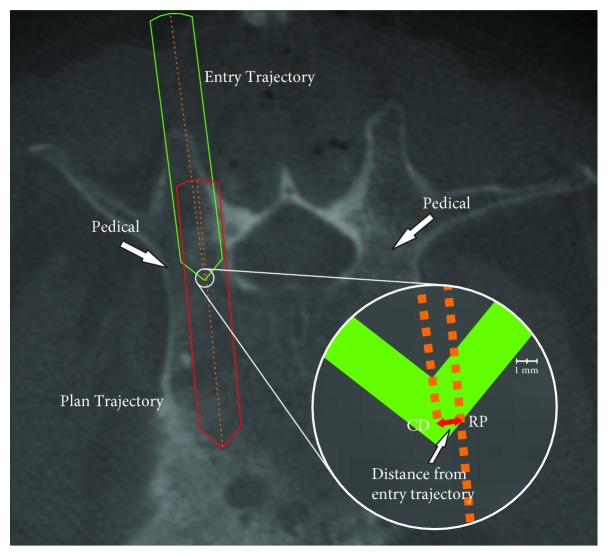
A screenshot taken from the StealthStation that illustrates the Entry vector (green) and Plan vector (red). Vectors have been outlined and center lines have been added to more clearly show actual deviations. The zoomed section shows the reference point (RP) as measured from the original terminal point of the entry vector.

Once point RP has been determined along the Plan trajectory, it is then used to compare with the Screw and Actual trajectories (Figure 7). Thus, the shortest distance between the Screw trajectory and RP and the Actual trajectory and RP can be calculated to determine the overall translational offset of these trajectories from RP in this critical region. While this calculation will not result in the deviation between RP and the Actual and Screw trajectories at exactly the depth within the pedicle as defined by the critical region, small angular and reference point deviations (<10 degrees and <5 mm, resp.) guarantee that even in the most extreme cases, this measurement will be taken at a depth of ±1 mm from the depth within the pedicle along the respective trajectory. The shortest distance from the Screw trajectory to RP and the Actual trajectory to RP can thus be used as a metric to determine accuracy with respect to the original Plan trajectory within this critical region of the pedicle isthmus.

In addition, because the Screw trajectory is also navigated, the final reference point deviation of the Actual trajectory from this additional reference point was also considered. While this trajectory is not shown in Figure 6, for calculation purposes, the Plan trajectory can simply be replaced with that of the Screw trajectory.

The distance from the marked reference point along the Plan trajectory to the Actual trajectory (i.e., the Euclidean distance) is calculated according to

(3) $$\begin{array}{c} d_{RP}=\frac{\left|\left(\alpha_{2}-\alpha_{1}\right)\times \left(\alpha_{1}-RP\right)\right|}{\left|\left(\alpha_{2}-\alpha_{1}\right)\right|}, \end{array}$$

where *α*_2_ and *α*_1_ represent the terminal point and entry point of the Actual trajectory, respectively. The orthogonal distance that is calculated is thus the shortest distance between the Reference Point and the Actual trajectory.

All computations were performed in MATLAB with reference points, trajectories, and distances drawn graphically to provide visual verification.

## 4. Results

Results are divided into two sections. The results of the angular deviation analysis are presented, followed by the results of the reference point analysis. It should be noted that unequal sample sizes between the two groups and skewed data distributions were anticipated (and observed) in the results of this study. For this reason, a permutation method was implemented to determine the *p* values shown in these results.

### 4.1. Angular Deviation

**Figure 8:**
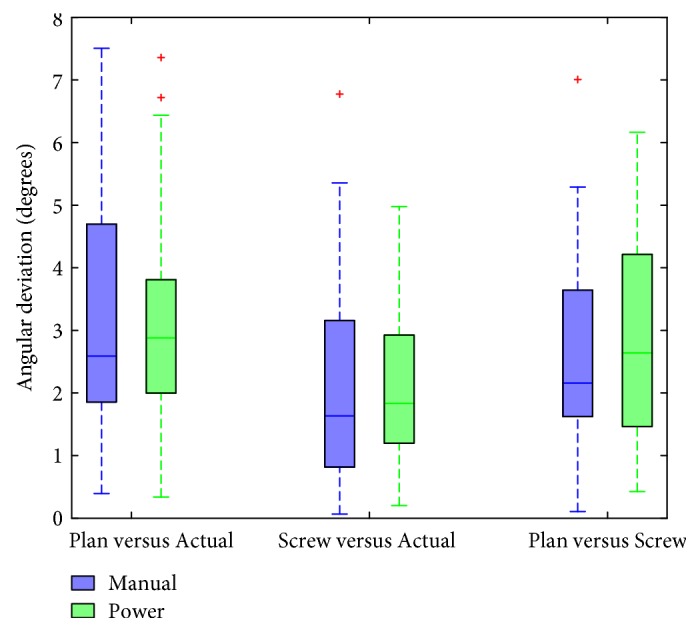
Angular deviations are shown for manual and power drivers. Median lines are shown within boxes; hash marks represent the outer quartile range. Outliers are shown in red. Mean values for the manual driver are 3.44, 2.13, and 2.94 degrees from left to right. Mean values for the power driver are 3.35, 2.08, and 2.88 degrees from left to right.

Angular deviations for all screws were calculated across both the manual and power groups. These results are shown in Figure 8. Overall, manually driven screws showed a higher mean angular deviation for all compared trajectories (Plan versus Actual, Screw versus Actual, and Plan versus Screw), when compared to the power-driven screws (3.44 degrees versus 3.35 degrees for the Plan versus Actual comparison). These differences, however, were not statistically significant for any of the comparisons between the manual and power drivers (*p* = 0.853, *p* = 0.856, *p* = 0.913 for Plan versus Actual, Screw versus Actual, and Plan versus Screw, resp.).

### 4.2. Reference Point Deviation

**Figure 9:**
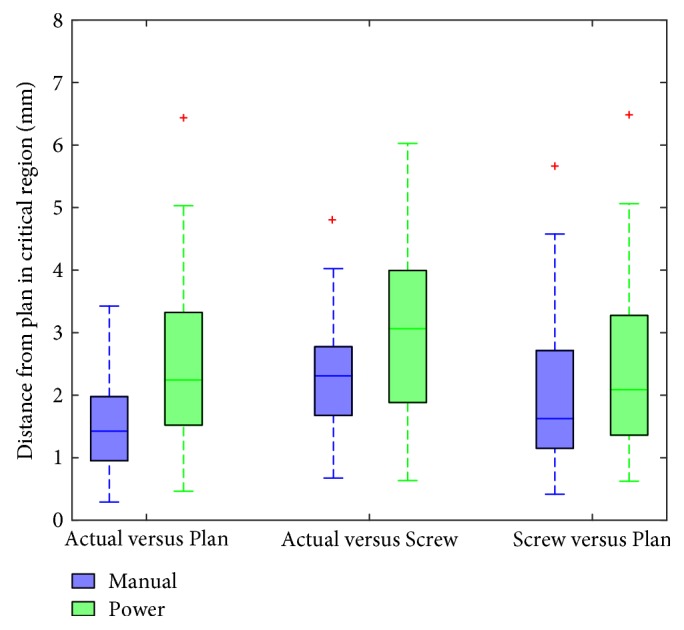
Reference point deviations are shown for manual and power drivers. Median lines are shown within boxes; hash marks represent the outer quartile range. Outliers are shown in red. Mean values for the manual driver are 1.54, 2.30, and 1.94 mm from left to right. Mean values for the power driver are 2.45, 3.05, and 2.26 mm from left to right.

Reference point deviations for all screws were calculated for both the manual and power groups. The results of this comparison are shown in Figure 9. Overall, the manual driver showed lower mean reference point deviations across all tested trajectories with mean values of 1.54 mm, 2.30 mm, and 1.94 mm for the manual driver and 2.45 mm, 3.05 mm, and 2.26 mm for the power driver. While this mean difference was relatively small, in contrast to the angular deviations, this difference was found to be statistically significant for the most important comparisons of the Actual versus Plan trajectories (*p* = 0.002) and Actual versus Screw trajectories (*p* = 0.009). While Screw versus Plan trajectories were not found to be statistically significant using this metric (*p* = 0.321), this was to be expected, as both of these trajectories are entirely dependent on the navigated tools and do not rely on the final position of the screw within the pedicle.

## 5. Conclusion

The results of this study suggest that there is some small performance lost via use of the power driver relative to the manual pedicle screw driver. While angular deviations from the Plan trajectory were statistically insignificant with means in favor of the power driver, the statistically significant translational errors in the critical region indicate that the power driver was less able to follow the Plan trajectory accurately through the pedicle isthmus, potentially increasing the risk of perforation in this region. However, in this study, no perforations were observed.

## 6. Discussion

This study presents a novel metric for assessing pedicle screw placement performance, utilizing a reference point within the pedicle to compare trajectories in 3D. While previous work has relied on the more simplistic review of X-rays and CT scans, to our knowledge, no previous work has attempted to use a metric such as reference point deviation in assessing screw placement.

The conclusions drawn from this study are based entirely on the metrics presented here. It should be noted however that this paper is not intended to present a risk–benefit analysis of the manual versus power drivers but rather to provide an analysis of two important performance metrics: angular deviation and reference point deviation.

While the reference point deviation metric has, to our knowledge, not been used previously, the angular deviation metric has been used in other studies to access the accuracy of pedicle screw placement using 3D navigation [19]. The mean values found for angular deviation in this study do fall within the range of values observed in these previous studies.

One method which is often cited to access successful screw placement is the Gertzbein-Robins criterion, which defines success based on the distance in mm by which a screw breaches the pedicle [20]. While this method is a useful qualitative approach for determining overall procedure success, the resolution of this method is not appropriate for accessing small performance differences between surgical tools.

Many factors may influence whether a surgeon chooses to use a particular tool, and as different tools may entail different risks and benefits, it is important to recognize the limitations of these tools with respect to specific patients and procedures. As noted previously, the power driver used in this study has a number of potential advantages that may offset some of the performance risks noted here. In particular, the projected, decrease in procedure time may offer overwhelming benefits to both the patient and the surgeon. The decreased risk of infection that often stems from shorter, minimally invasive procedures may, in effect, be a much more important factor than any observed decrease in critical region accuracy.

In addition, the long-term impact of this driver on the surgeon is an important factor to consider. Manual tools used in minimally invasive procedures often require intense physical exertion from the surgeon over long periods of time. This exertion can result in localized muscle fatigue in the short term, potentially impeding a surgeon's ability to perform procedures quickly and accurately. Perhaps most importantly, repeated exertion at the level required by these tools and procedures may lead to repetitive stress injuries to surgeons over time, further impeding their endurance in the operating room.

Improved ergonomics and decreased insertion time may thus offer significant benefits to the surgeon and patient; however, length of procedure and surgeon fatigue were in no way evaluated in this study. To our knowledge, no independently conducted study currently exists demonstrating these impacts with the power driver used here. Thus, despite the results of this study, further work is needed to determine whether the overall performance of the power driver significantly impacts patient outcomes for better or worse.
